# Effect of AM Fungi Inoculation on Litter Bacterial Community Characteristics under Heavy Metal Stress

**DOI:** 10.3390/microorganisms10020206

**Published:** 2022-01-19

**Authors:** Tong Jia, Yu Wang, Xiaoxia Liang, Tingyan Guo

**Affiliations:** Institute of Loess Plateau, Shanxi University, Taiyuan 030006, China; wyu202111@163.com (Y.W.); liangxiaoxia2020@163.com (X.L.); guotingyan_2018@163.com (T.G.)

**Keywords:** arbuscular mycorrhizal fungi, litter, bacterial community, heavy metal stress, *Imperata cylindrica*

## Abstract

Because microorganisms are the primary driving force behind litter decomposition, they play an important role in maintaining ecosystem material and chemical cycling. Arbuscular mycorrhizal (AM) fungi can improve host plant tolerance to various environmental stressors, making their application in mining area remediation important. In this study, litter from the dominant plant species (*Imperata cylindrica*) in a copper tailings mining area was selected as the experimental material. We conducted a greenhouse-based heavy metal stress experiment to investigate how AM fungi affect litter microbial community characteristics and key ecological factors. Results showed that AM fungi species, heavy metal treatments, and their combined interaction had significant impacts on litter pH. Additionally, enzyme activities in litter were significantly affected by interactions between AM fungi species and heavy metal contaminates. *Ralstonia* was significantly positively correlated to lead (Pb) content, indicating that *Ralstonia* had a certain tolerance to Pb pollution. Sucrase and urease activity were increased when plants were inoculated with *Rhizophagus irregularis* under Pb stress. Furthermore, *Microbacterium*, *Brevundimonas*, and *Pseudonocardia* all may play important roles in litter decomposition, while a certain tolerance was observed in *Kushneria* and *Roseivivax* to heavy metal pollution when plants were inoculated with *Glomus mosseae*. Results showed that AM fungi affected litter bacterial community structure and function by influencing plant litter properties. By exploring interactions between AM fungi and bacterial communities in plant litter under heavy metal stress, we will better understand associative processes that promote the cycling of soil organic matter and nutrients contaminated by non-ferrous metal tailings.

## 1. Introduction

Litter is the link that connects plants and soil [1]. Moreover, its decomposition encourages the development of soil organic matter. It also plays an important role in the formation of soil carbon (C) pools and the release of mineral elements as well as various types of nutrients. Additionally, the role that litter decomposition plays is also important for regulating plant growth processes, while affecting the net productivity of terrestrial ecosystems [2]. As a type of symbiotic (mycorrhizal) microorganism, arbuscular mycorrhizas (AM) species are directly associated with soil and plant root systems and play critical roles in the host plant litter decomposition process. Many studies have been conducted on how AM fungi species influence litter decomposition, but results have been generally inconsistent. A study on woody plant litter reported that a particular AM fungi species (*Rhizophagus irregularis*) was able to significantly reduce its litter burden [3]. Another study reported that AM fungi can promote organic C decomposition under high CO_2_ and N_2_ concentrations [4]. Moreover, two AM fungi species (*Glomus mosseae* and *Glomus claroideum*) have been shown to significantly reduce decomposition coefficients in the root litter of *Leymus chinensis* [5].

The mechanism by which AM fungi affect litter decomposition is still unclear. Most studies have concluded that AM fungi symbiosis is obligate in nature and is subsequently unable to uptake nutrition saprophytically. However, by influencing the properties of host plant litter or microbial communities, AM fungi increased the nutrient uptake from substrate to host plant, and they may interact with other soil organism (e.g., free living bacteria) to decompose litter [6,7]. Isotopic labelling has also shown that AM fungi can directly decompose litter [7], and this type of mycorrhiza can significantly increase the initial nitrogen (N) and phosphorus (P) content of alfalfa (*Medicago sativa*) root litter, while also increasing the decomposition rate of root litter [8]. Additionally, interactions between AM fungi and soil microbial communities are complex. Associated mycelial secretions have been shown to contain H^+^, OH^−^, and organic acids, which all affect the microbial community structure of the rhizosphere [6]. Moreover, one AM fungi species (*Funneliformis mosseae*) has been shown to inhibit the growth of soil fungi and significantly reduce its overall biomass [9]; it can also promote the enrichment of *Mycena* in soil. *Mycena* is a typical lignin-decomposing bacterium that promotes the decomposition of litter [10]. Therefore, any changes in the microbial community induced by AM fungi may subsequently alter the production of metabolites and, consequently, impact litter decomposition.

At present, the focus of most relevant studies has been on the relationships between AM fungi and soil microbial communities in natural ecosystems. Thus, associated interactions between AM fungi and litter microbial communities in contaminated ecosystems remain poorly understood. A previous study [11] reported on severe heavy metal pollution in a copper tailings dam in the southern region of Shanxi Province, China, and the role of the dominant plant species (*Imperata cylindrica*) was found to be important in the restoration of the plant community in this mining area. That study also showed that a large amount of *I. cylindrica* litter had accumulated at the end of plant growing season in this copper tailings area. Another study found that organic C content and changes to the litter decomposition process closely correlated to the adsorption and accumulation of metal elements in litter as well as to affiliated fixation and synergistic deposition in a heavy metal polluted environment [12]. Such findings can broaden our understanding of the influencing effects that AM fungi have on microbial litter communities as well as associative contributions to litter decomposition. 

To sum up, we hypothesized that AM fungi, heavy metal treatments, and their interaction would impact on the litter properties and enzyme activities, and different AM fungi would affect the microbial community structure and function of litters. This study can also improve the reparative status of degraded environments, namely, by exploring the role of AM fungi regarding nutrient cycling in copper mining areas subject to severe heavy metal pollution. For example, this study could select some available strains, which could be used for restoration in mining area copper tailings area in copper mining areas. It is of great significance to improve the ecological restoration efficiency of copper tailings areas.

## 2. Materials and Methods

### 2.1. Experimental Design

**Figure 1:**
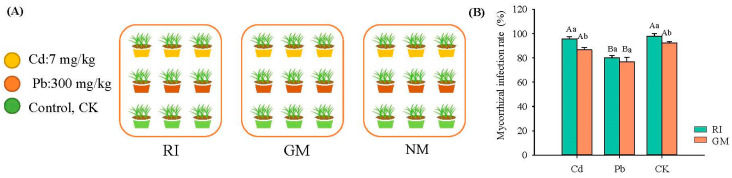
Experimental design (**A**) and infection rates of arbuscular mycorrhizal fungi (**B**) under different heavy metal stress. Note: Capital letters in figure (**B**) represent the difference between different heavy metal treatments in the same AM fungi inoculation. Lowercase letters represent the differences between different AM fungi inoculation under the same heavy metal treatment.

We conducted a fully-crossed (3 × 3) fractional experiment type design, applying three mycorrhizal treatments (RI: *Rhizophagus irregularis* inoculation treatment; GM: *Glomus mosseae* inoculation treatment; NM: non-inoculated mycorrhizal treatment) and three heavy metal treatments (cadmium (Cd): 7 mg/kg; lead (Pb): 300 mg/kg; CK, no-heavy metal [control] treatment) (Figure 1A). Here, the heavy metal treatment depended on the background values of soil heavy metals in copper tailings dam (Cd: 3.22–7.26 mg · kg^−1^; Pb: 176.36–287.88 mg · kg^−1^). Three replicates were made per treatment. The size of the pot was 14 cm high and 23 cm in diameter. We used 2 kg soil per plot for the plants, and inoculated with 100g AM inoculum in each plot in order to ensure they had the same number of spores. We selected *I. cylindrica* as the experimental plant material, which has a known high tolerance to heavy metals. We initiated heavy metal stress four weeks after plant seeds were sown, providing the time required for AM fungi inoculation to take place. For the heavy metal stress treatments (i.e., the Cd and Pb treatments), 3 CdSO_4_ · 8 H_2_O and PbSO4 was used, respectively. The duration of the experiment was 120 d, namely, from June 2020 to September 2020. This experiment was conducted in a greenhouse under temperatures ranging from 20 °C to 25 °C with a relative humidity ranging from 40–50% under natural daylight conditions. Each week, 100 mL of a half-strength Hoagland (nutrient) solution was added to each pot. Litters were collected in each pot at the end of plant growth. Pots were randomly repositioned every week to minimize any localized effects.

### 2.2. AM Fungus and Inoculation Procedure

The original AM fungus specimens were obtained from the Institute of Plant Nutrition and Resources, Beijing Academy of Agriculture and Forestry Sciences (BAAFS). Broomcorn (*Sorghum bicolor*) roots were placed in pots to culture the fungi over a 12-week period, after which the fungi were used to generate a large amount of inoculum for our experiment. The mycorrhizal inoculum contained a mixture of fungal spores, hypha, and root fragments. For the GM and RI treatments, vermiculite was mixed with 100 g of the mycorrhizal inoculum in each pot. For the NM treatment, we added the identical amount of the autoclaved inoculum as well as 50 mL of the washed non-autoclaved inoculum filtrate (sieved through a 10-μm mesh) to correct for potential bacterial and fungal community differences among the GM, RI, and NM treatments. Plants had relatively high infection rates. For RI inoculation treatment, the mycorrhizal infection rates were 96.7%, 80.0%, and 97.8% in Cd, Pb, and CK groups, respectively. For GM inoculation treatment, the mycorrhizal infection rates were 86.7%, 76.7%, and 92.2% in Cd, Pb, and CK groups, respectively (Figure 1B).

### 2.3. Litter Chemical Properties and Enzyme Activity

We collect the fresh litters on the surface at the end of plant growth seasons. Following this, fresh litter weight was measured. Portions of samples were dried at 65 ℃ to a constant weight, and the litter water content was then measured. An elemental analyzer (vario EL/MACRO cube, Elementar, Hanau, Germany) was used to measure total carbon (TC) and total nitrogen (TN) in litter samples. Litter water suspensions (1:20 mass/volume) were then shaken for 30 min prior to measuring litter pH [13]. Heavy metal (i.e., Cu, Zn, Pb, and Cd) concentrations in litter were measured using atomic absorption spectrometry (AAS) (Agilent Technologies 200 Series AA, Palo Alto, CA, USA). Additionally, 3,5-Dinitrosalicylic acid colorimetry was used to measure litter sucrase and cellulase content; phenol-sodium hypochlorite colorimetry was used to measure urease content; potassium permanganate titration was used to measure catalase content; the disodium phenyl phosphate colorimetric method was used to measure phosphatase content [11].

### 2.4. DNA Extraction, PCR Amplification, and Miseq Sequencing

We washed litter samples three times in sterile phosphate buffer solution (PBS: NaCl, KCl, Na_2_HPO_4_, and KH_2_PO_4_) prior to filtering through a sterile membrane filter (0.2 μm pore size) (Millipore, Jinteng, Tianjin, China). Sterile centrifuge tubes were used to seal filtered samples that were then used to extract microbial DNA. We used the E.Z.N.A.^®^ Soil DNA Kit (Omega Bio-Tek Inc, Norcross, GA, USA) to extract microbial DNA from litter. We also used the NanoDrop ND-1000 UV-Vis Spectrophotometer (NanoDrop Technologies, Wilmington, DE, USA) to quantify the extracted DNA. Primers 799F (5′-AACMGGATTAGATACCCKG-3′) and 1193R (5′-ACGTCATCCCCACCTTCC-3′) were used to amplify the V5–V7 hyper variable region of the 16S rRNA bacterial gene. The PCR mixtures contain 5 × *TransStart* FastPfu buffer 4 μL, 2.5 mM dNTPs 2 μL, forward primer (5 μM) 0.8 μL, reverse primer (5 μM) 0.8 μL, *TransStart* FastPfu DNA Polymerase 0.4 μL, template DNA 10 ng, and finally ddH_2_O up to 20 μL. PCR reactions were performed in triplicate. The PCR product was extracted from 2% agarose gel and purified using the AxyPrep DNA Gel Extraction Kit (Axygen Biosciences, Union City, CA, USA) according to manufacturer’s instructions and quantified using Quantus™ Fluorometer (Promega, Madison, WI, USA). Sequencing was performed at Shanghai Majorbio Bio-pharm Technology (Shanghai, China), employing the MiSeq platform (Illumina, Inc., San Diego, CA, USA). We submitted raw sequencing data to the National Center for Biotechnology Information (NCBI) Sequence Read Archive (SRA) (https://www.ncbi.nlm.nih.gov/sra (accessed on 18 December 2020)) under the project accession number PRJNA756117.

### 2.5. Sequencing Data Processing

QIIME was used to integrate the original FASTQ format sequencing data [14]. Usearch (ver. 7) was used to investigate and extirpate chimeric sequences (http://drive5.com/usearch/ (accessed on 14 October 2020)). We identified 97% sequence similarity as the operational taxonomic unit (OTU) partition threshold for classification results, which we subsequently used to determine bacterial community diversity and relative abundance. To obtain classification information on each species that corresponded to each OTU, the RDP classifier (https://rdp.cme.msu.edu/classifier/classifier.jsp (accessed on 14 October 2020)) was used to classify and analyze each OTU sequence. The SILVA database was used to compare each bacterial sequence, with a reliability threshold of 70%.

### 2.6. Statistical Analysis

SPSS 24.0 was used for the analysis of physical and chemical properties in litter. Duncan’s multiple range test was used in one-way ANOVA. A Student’s *t*-test was used to compare both bacterial diversity and richness in litter. LEfSe analysis was used to analyze differences between bacterial community composition for the different treatments. Linear discriminant analysis (LDA) was used to ascertain whether bacteria significantly influenced sampling division differences, with an LDA threshold of 2. In the construction of molecular ecological networks of bacterial phylogeny for the different AM fungal treatments, we used the random-matrix theory (RMT) at a genus level to identify any interactions among microbial communities. We obtained network parameters from the Molecular Ecological Network Analyses Pipeline (http://ieg4.rccc.ou.edu/mena (accessed on 15 October 2020)) website [15]. PICRUSt2 was employed for predicting litter bacterial community functions based on the Kyoto Encyclopedia of Genes and Genomes (KEGG) database.

## 3. Results

### 3.1. AM Fungi Effects on Litter Properties and Enzyme Activities

Results showed that AM fungi inoculation, heavy metal treatments, and their combined interaction all significantly affected litter pH (*p* < 0.05; Table 1). Litter pH increased under the GM treatment in the absence of heavy metal stress, while litter pH decreased under the RI treatment. AM fungi inoculation can significantly reduce litter pH under Cd stress. However, litter pH significantly increased in the RI treatment and decreased in the GM treatment under Pb stress. Moreover, litter water content increased (*p* < 0.05) in the RI treatment under Cd stress (Table 2).

AM fungus inoculation, mycorrhiza, and combined heavy metal interactions all significantly influenced litter enzyme activities (Table 3). Moreover, AM fungi inoculation also increased urease activity significantly, decreased cellulase activity in the GM treatment, and decreased urease, sucrase, and cellulase activities significantly in the RI treatment. Litter urease, sucrase, and cellulase activities were all significantly higher in GM treatment than in the RI treatment (Table 4). Under Cd stress, urease and cellulase activities significantly decreased in the RI treatment. However, extracellular enzyme activity in litter significantly decreased in the GM treatment under Cd stress. Additionally, cellulase activity significantly decreased, but urease and sucrase activities both increased in the RI treatment under Pb stress. Although catalase activity decreased, urease, sucrase, and cellulase activities increased in the GM treatment under Pb stress (*p* < 0.05; Table 4).

### 3.2. Effects of AM Fungi on Litter Bacterial Community Compositions and Diversities

**Figure 2:**
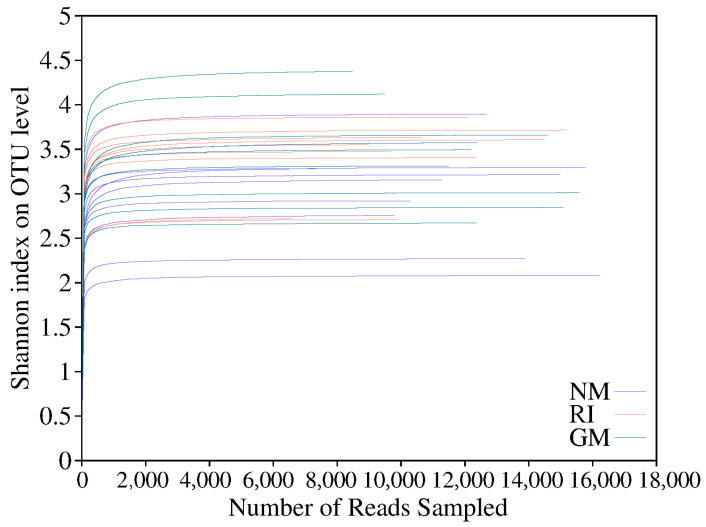
The rarefaction curves of bacterial community on OTU level under different heavy metal stress.

**Figure 3:**
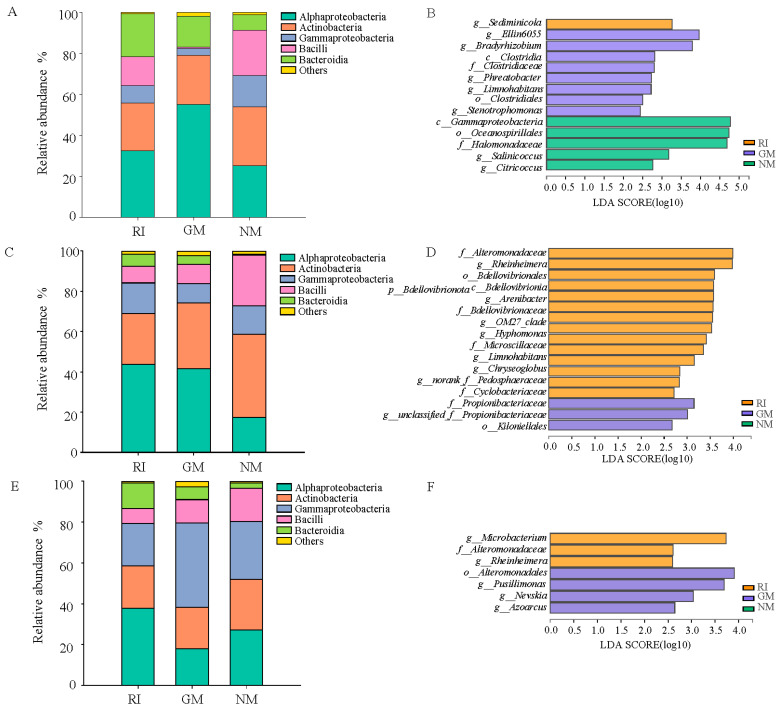
Bacterial community compositions at class level (**A**,**C**,**E**), and significantly different bacterial groups (**B**,**D**,**F**) in litters of *I. cylindrica* as influenced by AMF and heavy metal.

In total, we obtained approximately 507,613 sequence reads of the 16S rRNA gene with an average length of approximately 377 bp after trimming and chimera removal in all samples. We used a 97% similarity cutoff value to delineate OTUs in our downstream analyses. The coverage for the observed OTUs was 99% (Table 5) and the rarefaction curves showed clear asymptotes (Figure 2), which together indicate a near-complete sampling of the community. The dominant bacteria in litter under all treatments were Alphaproteobacteria and Actinobacteria. Gammaproteobacteria was also a dominant litter bacterium under heavy metal stress treatments (Figure 3A,C,E). Moreover, the relative abundances of Alphaproteobacteria and Bacteroidia increased under AM fungi inoculation in the control (CK) and Cd stress treatments (Figure 3A,C). The relative abundance reached 55.134% in the GM treatment under no heavy metal stress (CK). There was a significant decrease in the relative abundance of Gammaproteobacteria (*p* < 0.05). The relative abundance of Bacilli decreased after AM fungi inoculation, while the relative abundance of Bacteroidia increased under Pb stress. The RI treatment enriched litter Alphaproteobacteria in the RI treatment, while Gammaproteobacteria reached its highest relative abundance (41.12%) in the GM treatment under Pb stress (Figure 3E). It is important to note that we found no significant differences in either diversity or richness for the litter bacterial communities under all treatments (Figure 3B,D,F).

As confirmed by LEfSe analysis, AM fungi inoculation altered bacterial community composition in litter, particularly at genus level. In the absence of heavy metal stress, we observed extreme sensitivity in 14 bacterial groups in the RI, GM, and NM treatments (*p* < 0.05; LDA > 2). Sediminicola was significantly enriched in the RI treatment. A total of eight bacterial groups were enriched under the GM treatment, including *Ellin6055*, *Bradyrhizobium*, *Phreatobacter*, *Limnohabitans*, and *Stenotrophomonas* at a genus level (Figure 3B). *Salinicoccus* and *Citricoccus* were significantly enriched in the NM treatment (Figure 3B). Under Cd stress, we observed extreme sensitivity in 17 bacterial groups in the NM treatment (*p* < 0.05; LDA > 2). The relative abundances of *Rheinheimera*, *Arenibacter*, *OM27_clade*, *Hyphomonas*, *Limnohabitans*, and *Chryseoglobus* significantly increased in the RI treatment under Cd stress, while the relative abundance of Propionibacteriaceae increased in the GM treatment under Cd stress (Figure 3D). We observed extreme sensitivity in seven bacterial groups to Pb stress in the RI, GM, and NM treatments (*p* < 0.05; LDA > 2). Among these, *Microbacterium* and *Rheinheimera* were significantly enriched in the RI treatment, while *Pusillimonas*, *Nevskia*, and *Azoarcus* were significantly enriched in the GM treatment under Pb stress (Figure 3F).

We estimated Chao1 species richness indices were higher in GM treatment under different heavy metal treatments (Table 5). We found that GM under Pb stress had the highest ACE richness of all samples investigated in this study (Table 5). However, diversity indices, such as the Shannon and Simpson indices, showed different trends between the different treatments. Indicators of alpha diversity demonstrated that the bacterial community in litters was higher with GM inoculation compared to RI in CK treatment. On the other hand, the Simpson indices was significantly higher in NM treatment than AM fungi infection groups (Table 5).

### 3.3. Effects of Litter Properties and Enzyme Activities on Bacterial Communities for the Different AM Fungi Inoculation Treatments

**Figure 4:**
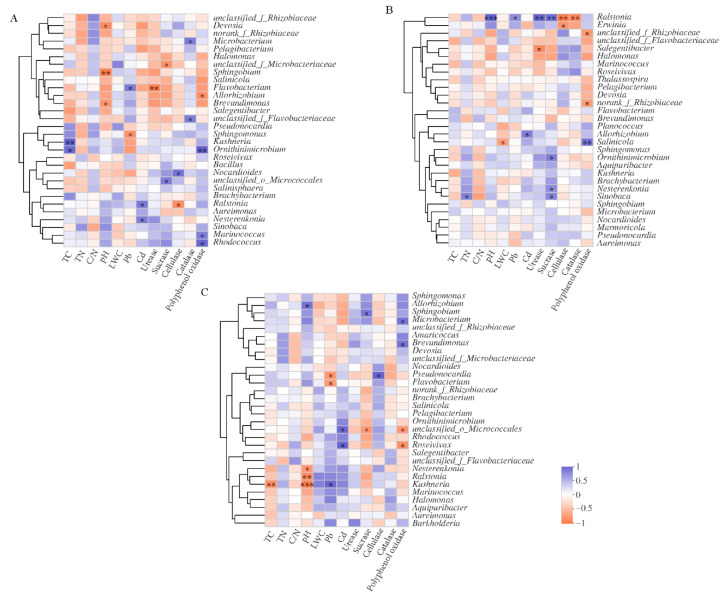
Correlation analysis of litter properties and dominant bacterial genera treated with RI (**A**), GM (**B**), and NM (**C**). Significance levels were denoted with * *p* < 0.05, ** *p* < 0.01 and *** *p* < 0.001.

Correlation analysis was conducted between the top 30 bacterial genera and the physical properties of litter (Figure 4). Results showed that *Marinococcus*, *Ornithinimicrobium*, and *Rhodococcus* were significantly and positively correlated to polyphenol oxidase activity in the NM treatment. *Microbacterium* and *Nocardioides* were positively correlated to both catalase and cellulase activity, respectively, in the NM treatment, indicating that these two microorganisms may be able to decompose litter. Additionally, *Nesterenkonia*, *Ralstonia*, and *Sinobaca* all significantly and positively correlated to Cd content, while *Flavobacterium* positively correlated to Pb content, indicating that these bacteria had a certain tolerance to heavy metal stress (Figure 4A).

*Salinicola* positively correlated to polyphenol oxidase activity in the RI treatment (*p* < 0.01), while *Nesterenkonia, Ornithinimicrobium,* and *Sinobaca* positively correlated to sucrase activity (*p* < 0.05). This indicated that these bacteria all played important roles in cellulose and lignin degradation in litter. Additionally, we found significant and positive correlations between *Allorhizobium* and Cd content as well as between *Ralstonia* and Pb content in the RI treatment. *Ralstonia* was significantly and positively correlated to sucrase and urease activities, indicating that *Ralstonia* may have a certain tolerance to Pb contamination and may potentially promote litter decomposition (Figure 4B). *Microbacterium* and *Brevundimonas* positively correlated to polyphenol oxidase, while *Pseudonocardia* positively correlated to cellulase in the GM treatment (*p* < 0.05). Additionally, *Kushneria* significantly correlated to Pb content in litter, and *Roseivivax* positively correlated to Cd content, indicating a potential tolerance of these microorganisms to heavy metal stress (Figure 4C).

### 3.4. Effects of AM Fungi Inoculation on the Functional Characteristics of Bacterial Communities in Litter

**Figure 5:**
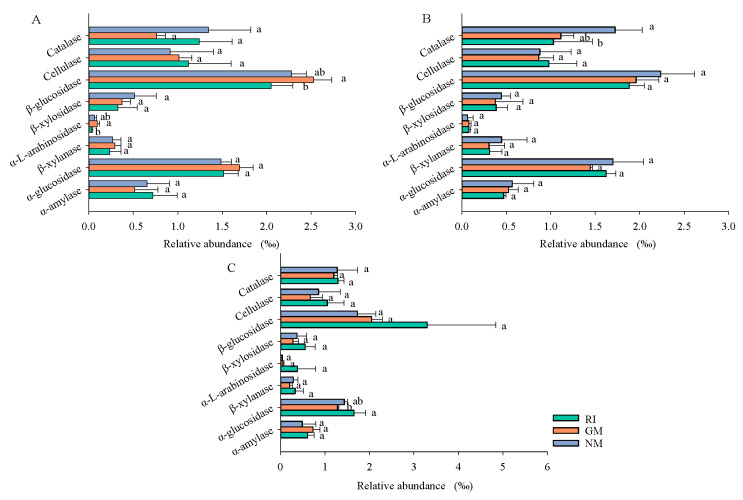
Effects of AM fungi on functional characteristics of bacterial community were predicted based on PICRUSt2 analysis under CK (**A**), Cd (**B**), and Pb (**C**) treatments. Different lowercase letters represent significant differences (*p* < 0.05).

Effects of AM fungi on functional characteristics of bacterial community were predicted based on PICRUSt2 (Figure 5). For the different treatments, gene encoding of cellulase, hemicellulase, and ligninase was observed in the bacterial communities (Figure 5), which indicated that bacterial communities played important roles in litter decomposition. The abundance of gene encoding β-glucosidase and α-l-arabinosidase in the bacterial community of the CK (control) treatment was significantly higher compared to the RI treatment (*p* < 0.05). This indicated that *G. mosseae* promoted the degradation of labile C in litter, and the decomposition effect was stronger than it was for *R. irregularis* in the CK treatment. AM fungi inoculation decreased the gene encoding abundance of cellulase, hemicellulase, and ligninase, and the decrease in the gene encoding abundance of catalase was particularly significant following AM fungi inoculation under Cd stress (*p* < 0.05). Results showed that AM fungi inoculation reduced the ability of bacterial communities to decompose litter under Cd stress, thus reducing litter decomposition rates. In Pb-contaminated soil, the relative abundance of α-glucosidase genes in litter was significantly higher in the RI treatment than in the GM treatment, indicating that the effect of *R. irregularis* on litter decomposition was stronger than that of *G. mosseae* under Pb-contaminated soil.

## 4. Discussion

### 4.1. Effects of AM Fungi on Litter Properties

Litter properties are important for litter decomposition, and it is generally believed that the decomposition rate of litter is inversely proportional to the C/N ratio [16]. Previous studies have found that AM fungi had no significant effect on antecedent aboveground litter content for *Leymus chinensis* and *M. sativa*, while no significant difference was observed in litter decomposition rates [5]. These findings were consistent with results from previous study. Heavy metal content also affected litter decomposition. It was reported that Pb can inhibit cellulase and urease activities in litter and subsequently effect the decomposition of *Phyllostachys pubescens* litter [17]. However, cellulase and urease activities were promoted after AM inoculation, which increased litter decomposition under Pb stress. Moreover, AM fungi has been shown to promote host plant growth and increase both aboveground and belowground plant biomass [18]. This ultimately causes a biological “dilution effect” which subsequently reduces the heavy metal burden of plants [18]. In this study, we found that AM fungi inoculation can significantly influence the litter pH. Previous studies showed that there have an indirectly plant driven observed between soil pH and microbial community structure [19]. Soil pH was closely correlated with bacterial diversity and soil microbial communities [20]. Similarly, there is a close relationship between AM fungi and litter pH, which could affect the microbial community of litters. 

### 4.2. Induced AM Fungi Alteration of Bacterial Community Composition in Litter

In this study, the inoculation of AM fungi and heavy metal stress altered the bacterial community composition in host plant litter. Specifically, the AM fungal inoculation treatments increased the relative abundance of both Alphaproteobacteria and Bacteroidia. Moreover, AM fungi can cause a significant correlation between Alphaproteobacteria and ligninase activity for various cellulase types [21]. Alphaproteobacteria consists of many genes that can encode lignocellulolytic enzymes, which indicated that Alphaproteobacteria can synthesize a variety of lignin and cellulase enzymes [22]. Furthermore, AM fungi inoculation significantly enriched *Bradyrhizobium, Microbacterium, Ellin6055, Rheinheimera,* and *Limnohabitans*. Among these bacterial genera, *Bradyrhizobium* and *Microbacterium* are two typical lignin-decomposing bacteria [23]. Furthermore, our study found that *Microbacterium* was significantly correlated to both catalase and polyphenol oxidase activity. It has been reported that the role that *Rheinheimera* plays in organic C decomposition is important [24]. These results suggested that AM fungi inoculation may attract and promote microbial groups associated with litter decomposition to colonize litter, subsequently promoting litter decomposition. Additionally, *Ornithinimicrobium, Nesterenkonia*, and *Sinobaca* significantly correlated to sucrase, cellulase, and catalase activities while exhibiting a certain tolerance to heavy metals (Cd, Pb, etc.). Previous studies showed that bacteria had effects on heavy metal remediation. AM fungi indirectly affect the heavy metal speciation by changing the microbial community structure and physicochemical properties of rhizosphere soil [25]. AMF strains isolated from different contaminated sites tended to have different tolerance ranges to heavy metals [26,27]. Moreover, AM fungi changed the heavy metal oxidizing and bacterial community structure in rhizosphere soil, and reduced the bioavailability of heavy metals [28]. Results therefore expounded the significance of these bacteria of *I. cylindrica* litter in heavy metal polluted areas.

### 4.3. Effects of AM Fungi on the Functional Characteristics of Bacterial Communities in Litter

In this study, we found that the inoculation of AM fungi would decrease the abundance of genes that encode catalase activity within litter bacterial communities, which was consistent with catalase activity trends. This indicated that AM fungi inoculation may reduce the ability of bacterial communities to decompose recalcitrant C sources, such as lignin. Moreover, AM fungi inoculation can increase the abundance of genes that encode cellulase and hemicellulase. The differences found between functional gene abundance and gene expression could have potentially resulted from activities associated with other microbial groups, except for bacteria during litter decomposition, such as fungi and protists. It was also reported that Basidiomycota members have the ability to secrete different varieties of cellulase and ligninolytic enzymes, which have a regulatory effect on litter decomposition [29]. Additionally, the role of oomycota is also important in the decomposition of organic matter [30]. Functional gene distribution can only predict the metabolic potential and the ecological function of bacterial communities; however, it is unable to predict actual metabolic conditions and the functional characteristics of bacterial communities [31]. Furthermore, a few other studies have also found differences between gene abundance and gene expression [32,33] Thus, it is important that we further investigate ways in which AM fungi effect functional characteristics of bacterial communities in combination with transcriptome, proteome, and other associated regulatory mechanisms.

## 5. Conclusions

Arbuscular mycorrhizal fungi play an important role in mining remediation. AM fungi had an effect on the microbial community characteristics of litters by influencing litter properties and enzyme activities, especially some key microbial groups with potential for heavy metal remediation. In the future, more attention should be paid to the dynamics of microbial community during litter decomposition, so as to deepen the understanding of the association between contaminated soil and nutrient cycling in mining areas.

## Figures and Tables

**Table 1:** The Two-way ANOVA analysis of heavy metal stress and AM fungal infection on litter properties.

	TC %		TN %		C/N		pH		LWC %		Pb mg/kg		Cd mg/kg	
	*F*	*p*	*F*	*p*	*F*	*p*	*F*	*p*	*F*	*p*	*F*	*p*	*F*	*p*
AMF	0.192	0.827	2.860	0.083	2.187	0.141	10.593	**0.001**	9.583	**0.001**	2.256	0.134	0.065	0.937
HM	0.647	0.535	1.075	0.362	1.434	0.264	281.336	**<0.001**	4.853	**0.021**	17.561	**<0.001**	17.648	**<0.001**
AMF × HM	0.576	0.684	0.343	0.845	0.352	0.839	487.540	**<0.001**	0.438	0.780	2.118	0.121	0.076	0.989

Note: Bold font indicates significant difference (*p* < 0.05). AMF represents AM fungal treatment, and HM represents heavy metal treatment. Abbreviations are as follows: mean total nitrogen (TN), total carbon (TC), the carbon and nitrogen ratio (C/N), litter water content (LWC), lead (Pb), and cadmium (Cd).

**Table 2:** Influence of AM fungi infection on the properties of litter under heavy metal stress.

Heavy Metal	AMF	TC %	TN %	C/N	pH	LWC %	Pb mg/kg	Cd mg/kg
CK	RI	37.164 ± 0.962 a	0.283 ± 0.011 a	131.377 ± 8.380 a	7.127 ± 0.006 c	0.314 ± 0.013 a	1.174 ± 0.501 a	0.093 ± 0.021 a
	GM	37.618 ± 0.619 a	0.313 ± 0.054 a	123.044 ± 24.201 a	7.503 ± 0.007 a	0.208 ± 0.042 a	1.273 ± 1.029 a	0.045 ± 0.014 b
	NM	37.253 ± 1.008 a	0.349 ± 0.081 a	110.717 ± 26.098 a	7.273 ± 0.049 b	0.204 ± 0.095 a	0.699 ± 0.191 a	0.034 ± 0.018 b
Cd	RI	36.323 ± 1.221 a	0.344 ± 0.066 a	108.508 ± 21.993 a	7.427 ± 0.015 b	0.273 ± 0.016 a	1.387 ± 0.531 a	5.095 ± 4.067 a
	GM	37.341 ± 0.476 a	0.294 ± 0.017 a	127.318 ± 9.121 a	7.437 ± 0.015 b	0.209 ± 0.038 b	1.1445 ± 0.458 a	6.314 ± 4.060 a
	NM	37.076 ± 1.876 a	0.460 ± 0.180 a	88.877 ± 31.706 a	7.723 ± 0.015 a	0.181 ± 0.031 b	1.292 ± 1.172 a	6.000 ± 4.132 a
Pb	RI	36.874 ± 2.335 a	0.359 ± 0.104 a	109.804 ± 37.580 a	7.743 ± 0.006 a	0.323 ± 0.055 a	9.647 ± 11.226 a	0.114 ± 0.057 a
	GM	35.935 ± 1.397 a	0.338 ± 0.020 a	106.887 ± 10.263 a	7.247 ± 0.015 c	0.287 ± 0.004 a	19.306 ± 6.935 a	0.121 ± 0.014 a
	NM	37.166 ± 0.331 a	0.439 ± 0.166 a	92.001 ± 29.745 a	7.310 ± 0.017 b	0.251 ± 0.047 a	6.837 ± 1.012 a	0.077 ± 0.053 a

Note: Data were means ± standard deviation. Significant differences between sites (*p* < 0.05) were represented by letters (a > b > c).

**Table 3:** The Two-way ANOVA analysis of heavy metal stress and AMF infection on the enzyme activities of litter.

	Urease		Sucrase		Cellulase		Catalase		Polyphenol Oxidase	
	(mg · (g · 24 h)^−1^)		(mg · (g · 24 h)^−1^)		(mg · (g · 72 h)^−1^)		(mg · (g · 20 min)^−1^)		(mL · g^−1^)	
	*F*	*p*	*F*	*p*	*F*	*p*	*F*	*p*	*F*	*p*
AMF	136.789	<0.001	115.465	<0.001	69.487	<0.001	5.258	0.016	409.383	<0.001
HM	4.871	0.02	44.154	<0.001	0.283	0.757	0.516	0.605	170.613	<0.001
AMF × HM	406.132	<0.001	460.762	<0.001	3.777	0.021	3.032	0.045	42.865	<0.001

**Table 4:** Influence of AMF infection on the enzyme activities of litter under heavy metal stress.

Heavy Metal	AMF	Urease (mg · (g · 24 h)^−1^)	Sucrase (mg · (g · 24 h)^−1^)	Cellulase (mg · (g · 72 h)^−1^)	Catalase (mg · (g · 20 min)^−1^)	Polyphenol Oxidase (mL · g^−1^)
CK	RI	1.277 ± 0.132 c	1.844 ± 0.055 b	0.331 ± 0.005 c	6.628 ± 0.133 a	5.333 ± 1.155 a
	GM	2.663 ± 0.149 a	3.351 ± 0.019 a	0.415 ± 0.013 b	1.464 ± 0.934 b	5.667 ± 0.577 a
	NM	2.156 ± 0.055 b	3.326 ± 0.091 a	0.616 ± 0.027 a	6.165 ± 0.963 a	4.667 ± 0.577 a
Cd	RI	1.430 ± 0.035 c	3.044 ± 0.045 a	0.202 ± 0.0243 c	6.165 ± 1.164 a	6.667 ± 0.577 a
	GM	0.564 ± 0.080 b	1.212 ± 0.076 b	0.469 ± 0.023 b	1.310 ± 0.133 b	3.667 ± 0.577 c
	NM	4.052 ± 0.202 a	3.253 ± 0.159 a	0.543 ± 0.014 a	6.320 ± 1.164 a	5.333 ± 0.577 b
Pb	RI	1.770 ± 0.058 b	3.104 ± 0.072 a	0.051 ± 0.030 c	4.393 ± 0.000 a	6.000 ± 1.000 a
	GM	2.779 ± 0.174 a	2.734 ± 0.048 b	0.417 ± 0.025 a	2.697 ± 1.273 b	5.000 ± 1.000 a
	NM	1.066 ± 0.063 c	2.397 ± 0.057 c	0.310 ± 0.033 b	6.242 ± 0.801 a	3.333 ± 2.309 a

Note: Data were means ± standard deviation. Significant differences between sites (*p* < 0.05) were represented by letters (a > b > c).

**Table 5:** Comparison between coverage and diversity estimators of litter bacterial communities under different treatments.

Heavy Metal	AMF	Richness Estimator		Diversity Index		Coverage%
		ACE	Chao1	Shannon	Simpson	
CK	RI	236.820	243.000	3.410	0.095	99.212
	GM	341.680	355.170	3.526	0.075	98.793
	NM	306.840	280.550	3.048	0.150	98.982
Cd	RI	272.430	252.150	3.514	0.061	99.125
	GM	279.030	292.080	3.279	0.102	99.140
	NM	301.540	283.820	2.830	0.167	98.987
Pb	RI	294.250	279.180	3.251	0.097	98.997
	GM	388.420	357.910	3.486	0.099	98.654
	NM	311.510	277.340	3.060	0.121	98.910

## Data Availability

Not applicable.
